# A Hybrid Cuckoo Search and Differential Evolution Approach to Protein–Ligand Docking

**DOI:** 10.3390/ijms19103181

**Published:** 2018-10-15

**Authors:** Hang Lin, Shirley W. I. Siu

**Affiliations:** Department of Computer and Information Science, University of Macau, Macau, China; lhang33@126.com

**Keywords:** structure-based drug design, optimization, metaheuristic, cuckoo search, differential evolution, conformational search, AutoDock Vina, PSOVina, CuckooVina

## Abstract

Protein–ligand docking is a molecular modeling technique that is used to predict the conformation of a small molecular ligand at the binding pocket of a protein receptor. There are many protein–ligand docking tools, among which AutoDock Vina is the most popular open-source docking software. In recent years, there have been numerous attempts to optimize the search process in AutoDock Vina by means of heuristic optimization methods, such as genetic and particle swarm optimization algorithms. This study, for the first time, explores the use of cuckoo search (CS) to solve the protein–ligand docking problem. The result of this study is CuckooVina, an enhanced conformational search algorithm that hybridizes cuckoo search with differential evolution (DE). Extensive tests using two benchmark datasets, PDBbind 2012 and Astex Diverse set, show that CuckooVina improves the docking performances in terms of RMSD, binding affinity, and success rate compared to Vina though it requires about 9–15% more time to complete a run than Vina. CuckooVina predicts more accurate docking poses with higher binding affinities than PSOVina with similar success rates. CuckooVina’s slower convergence but higher accuracy suggest that it is better able to escape from local energy minima and improves the problem of premature convergence. As a summary, our results assure that the hybrid CS–DE process to continuously generate diverse solutions is a good strategy to maintain the proper balance between global and local exploitation required for the ligand conformational search.

## 1. Introduction

Protein–ligand docking is a computational method that is used to predict the binding pose of a small molecular ligand at the active site of a target protein. It has been routinely used in both academia and industry to screen for new potent compounds and subsequently, has been used for lead optimization. The most notable and successful application of protein–ligand docking and virtual screening is the discovery of inhibitors against the human immunodeficiency virus (HIV) protease for curing AIDS disease [1]. Over the past decades, many protein–ligand docking programs have already been developed to aid these studies. Among others, AutoDock, developed by Morris and co-workers [2], is the most popular open-source program. It encodes the configuration of a given ligand into a solution vector and repeatedly *mutates* values in the vector to identify the ligand configuration that gives the optimal binding energy. AutoDock was found to achieve an accuracy of up to  2 kcal/mol. When the experimental structure of the protein–ligand complex is known, one can compare the predicted ligand binding pose of the docking program to the experimental binding pose. A small difference between the two (usually taken to be <2 Å) indicates successful docking.

The ultimate goal of a docking program is to achieve high structural accuracy while minimizing the computational cost, which has always been a challenge. Exploring a proper way to use computational power more efficiently has encouraged the upgrade from AutoDock 4 [2] to AutoDock Vina [3]. For instance, AutoDock Vina (also named Vina here for short) has significantly improved accuracy in terms of the binding pose prediction compared to AutoDock 4 through the use of a new scoring function. This knowledge-based scoring function also makes Vina run faster than AutoDock 4 by two orders of magnitude [3]. In addition, AutoDock Vina has updated its minimization method to a quasi-Newton Broyden–Fletcher–Goldfarb–Shanno (BFGS) algorithm and employed multi-threading to parallelize and accelerate the docking process.

In recent years, plenty of nature-inspired metaheuristic algorithms have been invented to solve complex and highly nonlinear optimization problems. As a matter of fact, the protein–ligand docking can be regarded as an optimization problem where the list of values defining the ligand configuration represent the design variables to be optimized, and the scoring function is the objective function which minimizes the free energy of interactions between the protein and the ligand. In view of this, studies have incorporated metaheuristic algorithms for protein–ligand docking or structural alignment, such as simulated annealing (SA) [4], tabu search (TS) [5], and ant colony optimization (ACO) [6,7]. A number of variants based on AutoDock or AutoDock Vina were developed, in which the search algorithm is replaced by a metaheuristic algorithm. To name a few, SODOCK [8], PSO@AUTODOCK [9], FIPSDock [10], F*l*APCps [11], PSOVina [12,13], and ADHDock [14] have recently been published and have demonstrated superior performance over the basic AutoDock or AutoDock Vina methods. During the development of PSOVina, we realized that the main weakness of the original particle swarm optimization (PSO) algorithm is insufficient exploration of the search space. Since all particles in PSO follow the same rule, evolving under the influence of the single best particle, quick but premature convergence is hard to avoid.

In this study, our goal was to investigate other metaheuristic algorithms for solving the protein–ligand docking problem, in particular the ligand conformation generation strategy during the docking process. Bearing in mind the weakness of PSO, we designed and tested a new search algorithm which combines the cuckoo search (CS) and differential evolution (DE) algorithms in a novel way to enhance the search diversity and to escape from local minima. CS is also a swarm intelligence-based algorithm. It was proposed by Yang and Deb in 2009 [15] to solve difficult nonlinear optimization problems. Since its inception, it has been widely adopted in various computer science and engineering areas, such as pattern recognition, software testing, data fusion, networking, and job scheduling [16,17]. It is based on the aggressive brood reproductive strategy of cuckoo birds to increase their own population. CS is computationally more efficient and effective than PSO, as demonstrated in a performance comparison study using problem specific distance functions [18]. Its effectiveness is due to a good balance between local and global random search controlled by a switching parameter. On the other hand, differential evolution (DE) is a unique, non-nature inspired evolutionary algorithm proposed by Storn and Price in 1995 [19], the same year that PSO was proposed by Kennedy [20]. It is well-known for its simplicity and capability to solve a diverse range of problems. It is simple and robust in that the same results can be obtained consistently over many repeated runs. DE is described as a method that can perform *contour matching*, meaning that the evolution of DE solutions closely follows the contours of the objective function [21].

Using the advantages of both CS and DE, we proposed a novel hybrid CS–DE docking algorithm named CuckooVina. It performs global exploration over the large conformational space of a ligand and simultaneously maintains good local exploitation to fine-tune the binding pose of the ligand. Our search strategy is different from most of the aforementioned docking methods that two metaheuristic algorithms were used in the search process. The two metaheuristic algorithms did not perform the search independently but rather complementarily where CS explores locally in the promising regions and DE undergoes large jumps to seek for new promising regions.

We tested the performance of CuckooVina using two benchmark datasets, PDBbind 2012 and the Astex Diverse set, and compared it with its predecessor AutoDock Vina [3], which uses a Markov Chain Monte Carlo approach as a global search technique and PSOVina [12,13] which uses PSO. All three methods use the same energy minimization algorithm and scoring function, which are the original implementations of Vina.

## 2. Results

### 2.1. Docking Experiment Setup

Our proposed docking algorithm CuckooVina is described in detail in the Materials and Methods section. We compared the docking performances of CuckooVina to AutoDock Vina and PSOVina, namely, their root mean squared deviations (RMSDs), success rates, predicted binding affinities, run time, and convergence value. The run time was obtained using the system command time that measures the elapsed time from start to finish of the process. To determine convergence of the search process, energy of the best solution found was monitored. Once a stable solution is reached, i.e., no better solution is found from that point until the end of the docking process, the search is considered as converged. The proportion of the search process to reach convergence was computed as the ratio of energy evaluations reaching convergence and total number of energy evaluations. Furthermore, to understand the search behavior of CS–DE and PSO, we measured the sum of all pairwise Euclidean distances of search agents to estimate the time evolution of the space searched by these agents. We also monitored the solution update behavior brought about by the hybridization of CS and DE in the search process, to gain further insight into a better search strategy for docking problems. Finally, we evaluated the effects of the number of search agents and number of parallel threads on the docking results. As for the experiments, we note that for different methods the number of energy evaluations in each iteration is different. To ensure a fair comparison, we set the same number of maximum energy evaluations for CuckooVina, PSOVina, and Vina. We used the same number of search agents, i.e., 8, in both CuckooVina and PSOVina for most experiments, unless explicitly specified. Other docking parameters were conveniently taken from publications where the methods were introduced. A complete list of the parameters and values used are presented in Table 1. Due to the stochastic nature of these methods, each docking run, even those using the same method, may generate different final solutions. To address this problem, the docking of each complex was repeated 30 times.

### 2.2. Single-Thread Docking Experiment

In AutoDock Vina, multi-threaded docking is performed by default when the number of CPU cores available in the machine is more than 1. Vina automatically generates a number of threads equal to the number of cores. Each of the threads runs the search process in parallel but independently, where the best docking solutions found in each search are pooled together to generate the final solution list. Inherited from Vina, both CuckooVina and PSOVina do the same.

Although the docking solution is generally improved by using a multi-threaded approach, we argue that this way of pooling results together from independent runs is not beneficial for the study of the search algorithm in the docking program. Since each fresh-start search process performs re-initialization of the population, as long as more repeats are done, the search space will be more globally covered which usually yields better overall lowest energy solutions. For this reason, the ability of the search algorithm to globally explore the search space cannot be fully revealed. Moreover, a search process which has been trapped in a local minima leading to premature convergence cannot be easily identified. Therefore, we employed a more rigorous experimental approach, i.e., to perform a single-thread docking experiment. Repeated dockings were mainly used to obtain statistics and examine the consistency of the search results.

**Figure 1:**
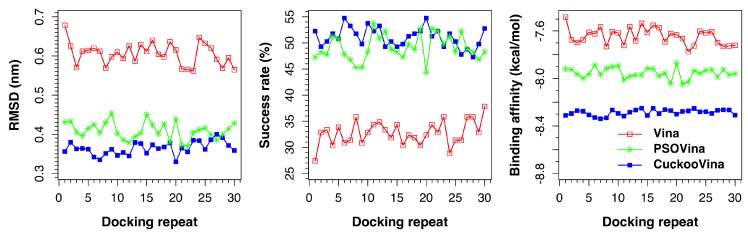
Docking performances of AutoDock Vina, PSOVina, and CuckooVina in 30 independent docking repeats using the PDBbind 2012 dataset. Each data point is an average of 201 complexes.

Figure 1 presents the results of all docking repeats on the PDBbind 2012 dataset. Each point represents the average value of all 201 complexes in one docking experiment. Five metrics were measured: RMSD, success rate, binding affinity, run time, and convergence value. A docking is considered successful if the docked ligand has an RMSD of <0.2 nm from the experimental ligand with respect to the protein. When the performances of three docking methods are compared, it is clearly seen that CuckooVina performs consistently better than both Autodock Vina and PSOVina in RMSD, success rate, and binding affinity (based on the post-hoc test of Tukey’s Honestly Significant Difference (HSD) using α = 0.05). It is worth mentioning that CuckooVina not only performs better, but the generated solutions are also more consistent over different independent runs than those of PSOVina and AutoDock Vina as observed from the low deviations in all three metrics among different docking repeats. As a consequence, the requirement of performing many docking repeats to obtain reliable results for other stochastic search methods may be relaxed for CuckooVina.

The average docking performances of three methods using the PDBbind 2012 dataset are summarized in Table 2. CuckooVina gives improvements over PSOVina of 11%, 4%, and 4% in terms of the RMSD, success rate, and binding affinity respectively (*p*-values of 8.26 × $10^{-10}$, 0.0049, 1.490 × $10^{-11}$, respectively, using Tukey’s HSD test at α = 0.05). As both PSOVina and CuckooVina have some overheads for managing search agents, they need about 39% and 15% more run time, respectively, to complete the same number of energy evaluations as Vina. To assess convergence speed, we computed the ratio of energy evaluations reaching a stable solution (i.e., no more updates until end of the docking process) to the total number of energy evaluations. Vina and PSOVina obtained convergence values of about 0.5, whereas CuckooVina obtained 0.69. Although Vina and PSOVina are converged faster, their lower performances in the success rate and binding affinity suggest that both of them were trapped in local energy minima that led to premature convergences. Using additional energy evaluations, solutions in CuckooVina continued to be optimized reaching closer to global minimum.

**Figure 2:**
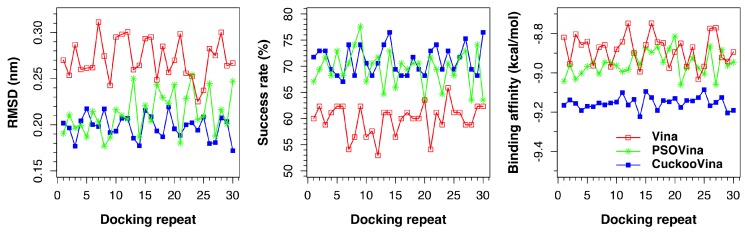
Docking performances of AutoDock Vina, PSOVina, and CuckooVina in 30 independent docking repeats using the Astex Diverse dataset. Each data point is an average of 85 complexes.

The same benchmarking exercise was performed using another docking dataset, the Astex Diverse dataset. As shown in Figure 2 and Table 3, the three docking methods exhibit almost the same order of performance as for the PDBbind 2012 dataset. Overall, CuckooVina still gives the best docking results in terms of lower RMSD (*p*-value of 1.49 × 10${}^{-11}$ versus Vina and 0.0172 versus PSOVina) and better binding affinity (*p*-value of 1.49 × 10${}^{-10}$ versus both Vina and PSOVina), though the difference in success rates between CuckooVina and PSOVina is insignificant (*p*-value = 0.138). We note that the deviations of CuckooVina are still the smallest among the three methods and it took about 9% more time than Vina to complete a docking run.

As a summary, the single-thread experiments assure that CuckooVina, which employs the hybrid CS and DE approach, shows superior performance than Autodock Vina. While having a similar success rate to PSOVina, CuckooVina predicts ligand binding poses more accurately with higher binding affinities.

### 2.3. Search Space Coverage

In our docking experiments, we used eight search agents in both CuckooVina and PSOVina. In PSOVina, all eight agents updated in the same manner, whereas in CuckooVina, the search agents updated differently depending on whether they were from the better set or from the worse set. How these search agents evolve is the core of the optimization algorithm which governs the efficiency of the search and accuracy of the result.

As the global minimum is generally unknown, the basic strategy to find this minimum is to search as widely as possible across the entire space for promising regions. On the other hand, the search cannot be performed uncontrollably; whenever promising regions are discovered, they should be sufficiently exploited to make sure that the global minimum is not missed. Since AutoDock Vina has a rather good built-in minimization method, the local minimum can usually be found with all three docking methods. Here, our focus was on the global exploration of the search space and the comparison of search behavior between the proposed hybrid CS–DE and PSO algorithms.

**Figure 3:**
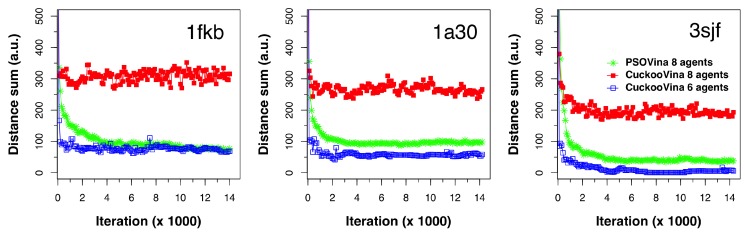
Search space coverage measured as the sum of pairwise distances between search agents in the docking process of the 1fkb, 1a30, and 3sjf complexes.

As a measure of search space coverage, we computed the *distance sum* (DS), the sum of pairwise Euclidean distances between search agents, as defined in Equation (1), at every 100 energy evaluation interval in the course of docking. We ran this experiment on dozens of protein–ligand pairs and selected three typical cases (1kfb, 1a30, and 3sjf) for illustration, as shown in Figure 3. Each of the subplots shows the DS evolution for the two methods. For CuckooVina, the results for both eight agents and the six agents from the better-set are shown. For better visual comparison, the DS values for six agents were multiplied by a factor ($m={}_{8}C_{2}/{}_{6}C_{2}$) to approximate their values as if there were eight agents.

The experiments were run with eight agents and the switching parameter was set to $P_{worse}=0.25$. Therefore, the better set contained six search agents and the worse set contained two. We can see that these six agents in CuckooVina maintained a small search space coverage exhibited by small DS values. When all eight agents were considered, the DS was greatly increased, revealing that the worse set agents explored the search space widely. This “wide-scope” search continued throughout the entire process.

In contrast, the search space coverage of the PSO with eight agents kept dropping from the start of the search process. In a low-dimensional space, we would consider this to be a good feature because it would search a wider space in the beginning and then converge to a rather small space to obtain a final answer. However, with a high-dimensional space as that in the protein–ligand docking problem, PSO’s quick collapse in search space coverage leads to the premature convergence problem. By maintaining a wide search scope throughout the search process, CuckooVina can locate the best energy poses while PSOVina and Vina were trapped in local minima.

### 2.4. Monitoring the Updating Situation of the Better and Worse Sets

To understand how Cuckoo Search overcomes the premature convergence problem, we closely monitored how the better and worse sets updated during the search process. An update in the better set was considered important when a newly generated solution had a binding affinity with a lower energy than the worst nest in the better set. Similarly, an update in the worse set was considered important when a newly generated solution in the modified DE process had a binding affinity with a lower energy than the worst nest in the better set. Whenever there was an important update, solutions were re-ranked, and a new and better solution was then included in the better set.

**Figure 4:**
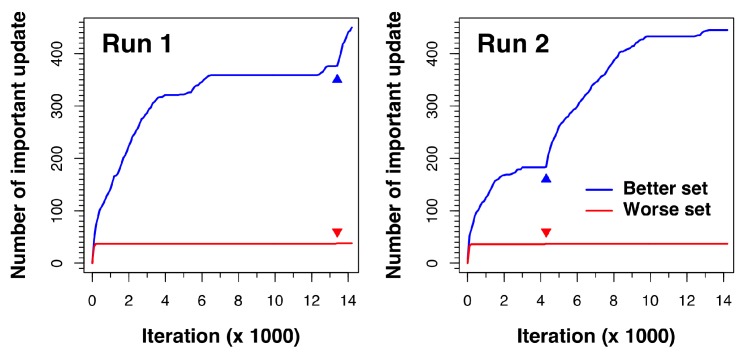
Number of updates that occurred in the better and worse sets in two docking runs of the representative complex 1a30. The colored triangles indicate the an iteration where an important worse set update (red) leads to subsequent rapid updates of the better set (blue).

Figure 4 shows how the docking process for complex 1a30 updates as the algorithm proceeds. The results of two independent runs are displayed. As shown in the figure, both sets (better and worse) update very rapidly in the beginning. However, after a few iterations, no new solutions from the modified DE process are better than those in the better set, so the worse set update line stays flat. In the meantime, the better set nests keep on improving via random walk in local regions until all the better set solutions converge to minima, where the better set update line also stays flat. The breakthrough moment comes when an important worse set update occurs (as indicated by a small red triangle in the figure). This new and better solution found by the modified DE process signals the discovery of a new potential low-energy region in the search space. The solutions are thus re-ranked and the new solution is placed in the better set. Once in the better set, exploration around the region of this newly discovered solution is conducted in later iterations, which is shown by the subsequent rapid rise of the better set update line.

### 2.5. Effect of the Number of Search Agents

**Figure 5:**
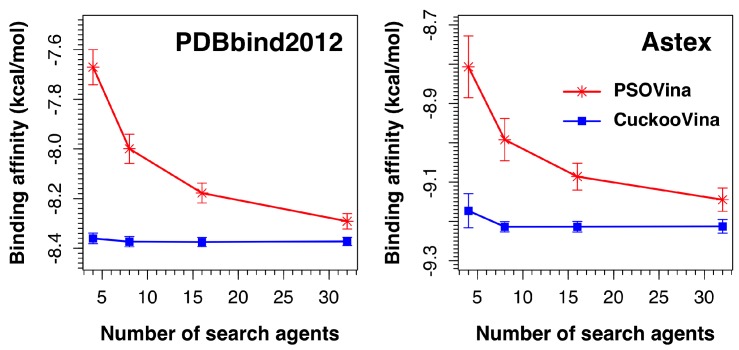
Effects of the number of search agents (4, 8, 16, and 32) on the docking performances of PSOVina and CuckooVina using the PDBbind 2012 and Astex datasets. Experiments were performed using 30 docking repeats.

We investigated the effect of the number of search agents by running experiments using 4, 8, 16, and 32 search agents. As shown in Figure 5, the performances of CuckooVina were minimally affected by the number of search agents in terms of binding affinity. On the contrary, the success of PSOVina critically depends on the selected number of search agents, typically, the more the better. In all cases, CuckooVina had smaller error bars than PSOVina, computed from 30 independent runs. This indicates that the docking algorithm of CuckooVina is more robust, and each docking result is more reliable.

### 2.6. Effect of the Number of Parallel Threads

AutoDock Vina supports the use of multiple threads to perform docking by specifying the exhaustiveness option. Each thread runs an independent ligand pose searching process and the found solutions are pooled together to select the top *k* results ranked by predicted binding affinities. Consequently, when more threads are used for docking, better solutions can be expected as more searches are performed. Both PSOVina and CuckooVina have inherited this feature from Vina, but, so far, our experiments have been done with a single thread to focus our analysis on the search algorithms. In this section, we describe an investigation on the effect of the number of threads on the docking performances by using 1–10 threads.

**Figure 6:**
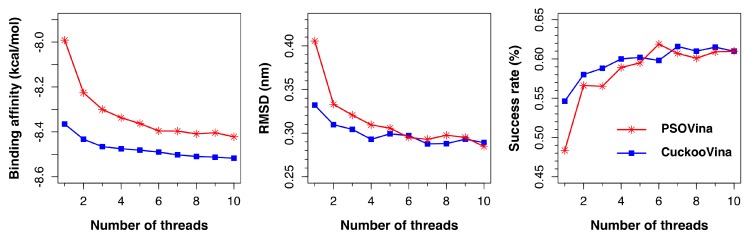
Effect of the number of threads on the docking performances of PSOVina and CuckooVina using the PDBbind2012 dataset.

As shown in Figure 6, both PSOVina and CuckooVina performed better with an increased number of threads, as expected. It is worth noting that, even with fewer threads, CuckooVina outperformed in all cases.

## 3. Materials and Methods

### 3.1. Cuckoo Search and Differential Evolution

Cuckoo search (CS) is a metaheuristic algorithm based on the parasitic breeding behavior of some cuckoo species. These cuckoos lay eggs in other birds’ nests. When the baby cuckoos hatch, which usually happens earlier than other chicks, they kick the others out of the nest and are then raised alone by the adoptive parents. Like other swarm-based methods, CS solves an optimization problem by maintaining a population of solutions and updating them in an iterative fashion. Here, a nest (or the egg) represents a *solution*, a cuckoo represents a *search agent*, and an egg-in-a-nest represents one of the solutions at the current iteration. The *replace* and *maintain* operations in CS were designed to mimic the aggressive behavior of cuckoos following three simple rules:

1. Each cuckoo lays one egg at a time and dumps it into a random nest (*first replace*).
2. Some proportion of nests that have the best eggs can carry them over to the next generations (*maintain*).
3. The fraction of remaining nests, those with the worst eggs, are abandoned by the host birds and new ones are built (*second replace*).

The first rule means that the search algorithm selects one nest for mutation and then compares the new solution to its existing solution. The second and third rules ensure that top solutions can carry on to the next generation while a proportion of not-so-good solutions are replaced by new ones. Yang introduced Lévy flight, providing a reasonably expensive exponential calculation, into CS [15] for the mutation step by performing a random walk with a step size that is proportional to the number of iterations.

The differential evolution (DE) has a similar pattern to other evolutionary algorithms: initialization, selection, mutation, and recombination. In each iteration, the DE algorithm first creates a donor vector, $x_{donor}=x_{1}-F\left(x_{2}-x_{3}\right)$, where $x_{k}=1,2,$ or 3 represents three randomly selected solutions from the current population, and *F* is the user-specified differential weight. A trial vector, $x_{trial}$, is then constructed by selecting each element of $x_{donor}$ for $x_{trial}$ with a probability of p∈[0,1]. Afterwards, $x_{trial}$ is compared with a randomly selected solution in the current generation. The better solution is kept for the next generation. These steps are repeated until a desirable population size is reached.

### 3.2. The Protein–Ligand Docking Search Problem

Our proposed optimization method for docking was integrated into AutoDock Vina [3]. This necessitated the search problem to first be described in Vina. In Vina, a ligand pose is represented by a vector of 7+τ elements, which includes the ligand position in Cartesian coordinates (3 elements), the ligand orientation in quaterion form (4 elements), and ligand rotatable torsions in single angular values (τ elements). The conformational search process is divided into two parts—global search and local search. The former is used to generate new ligand poses by randomly adjusting either the position, orientation, or one of the rotatable torsions of the ligand by some random amount. Each generated pose is refined to its closest minimum energy conformation through the quasi-Newton Broyden–Fletcher–Goldfarb–Shannon (BFGS) minimization algorithm. It is then subjected to Metropolis criteria to decide whether to keep or to revert back to the original pose. The main problem of the global search in Vina is that the random walk method does not utilize any previous knowledge of the search, except for the last pose. Since its step size and move direction are both generated randomly, Vina takes almost no advantage of the search history.

### 3.3. CuckooVina

In this work, we investigated the use of CS and DE to enhance the conformational search process in protein–ligand docking. Our implementation was based on AutoDock Vina. At first, we replaced the Markov chain Monte Carlo (MCMC) method with the original CS algorithm for the global search in Vina. However, two problems were observed: (1) The *first-replace* operation in CS, namely, randomly replacing any nest by a cuckoo’s offspring, limits the search diversity of the algorithm. Obviously, there is a higher chance that the cuckoo’s offspring, which has been mutated from a nest at a higher rank, can generate better eggs as compared to the eggs from a lower rank nest. This means that worse solutions are frequently replaced by better solutions from regions that have been explored, leading to premature convergence. (2) In the original CS algorithm, the Lévy flight method, which is used to the generate cuckoo’s offspring, is computationally expensive due to its exponential calculation. This makes the entire docking process very inefficient. As the ability to perform sufficient exploration of the conformational space is crucial to the success of protein–ligand docking, the CS algorithm needs to be improved such that it can search widely over the conformational space to avoid premature convergence and so that it is computationally less expensive.

Our ideas for such improvements were the following: We divided the host nests into two sets, the *better set* and the *worse set*. The former includes nests which have a high ranking among the list of nests and the latter are the remaining nests with worse solutions. During the *first-replace* operation, the egg in a random nest is selected to generate a new egg, and a nest from the  *better set* is then randomly selected to be considered for replacement. Here, the egg generation is performed using a random walk for efficiency. In the nest *second-replace* operation, each nest in the *worse set* is replaced by a new solution using a modified DE process. In this process, any two nests can be selected as templates to generate the donor vector. To ensure all solutions can be considered, each dimension in the solution vector is updated independently and is subject to some randomness. The individual updates take into account the width of the search space in the respective dimensions by virtue of the dimension-specific differential weights. By using these two different replace operations, newer solutions can be found. In addition, by carrying over the better solutions to the next generation, the quality of the solutions will be improved.

Our proposed CuckooVina algorithm is presented in Algorithm 1, and the modified DE process is presented in Algorithm 2. Three parameters are required. Two are the number of host nests (or the population size; *k*) and the switching parameter ($P_{worse}\in \left(0,1\right)$), which is the fraction of nests that are included in the worse set in the CuckooVina algorithm. Thus, $1-P_{worse}$ is the fraction of nests in the better set. The final parameter is the mutation probability ($P_{mutate}$) for the modified DE process.

**Algorithm 1:** CuckooVina for protein–ligand docking.
 1:Given the number of host nests (*k*) and the switching parameter ($P_{worse}\in \left(0,1\right)$) 2:Generate the initial population, *X* ← {$x_{1}$, …, $x_{k}$} 3:Compute the energy levels, $f\left(x_{1}\right),\ldots ,f\left(x_{k}\right)$ 4:Rank *X* according to the energy levels 5:Assign the $P_{worse}$ nests lower in rank as the *worse set* and the remaining nests as the *better set* 6:**while***t* < $t_{max}$ or stopping criteria **do** 7: Set a nest ($x_{i}$) randomly as the cuckoo 8: Generate its offspring ($x_{i}^{\prime }$) by a *random walk* 9: Compute $f(x_{i}^{\prime })$10: Choose a nest ($x_{j}$) randomly from the **better set**11: **if** $f\left(x_{i}^{\prime }\right)<f\left(x_{j}\right)$ **then**12:  $x_{j}\leftarrow x_{i}^{\prime }$13: **end if**14: Nests from the *worse set* are discarded, and new ones are built using the modified DE15: Re-rank *X*16: Update the *better set* and *worse set*17:
**end while**
18:Return solutions *X*


**Algorithm 2:** Modified DE for mutating a nest ($x_{w}$) in the *worse set*.
 1:Given the mutation probability ($P_{mutate}$) and the number of existing nests to be mutated ($x_{w}$) 2:Randomly choose two different nests, $x_{r}$ and $x_{s}$ 3:Compute the donor vector, $x_{d}=x_{r}-x_{s}$ 4:Initialize differential weights $\alpha_{p}$, $\alpha_{o}$, $\alpha_{t}$ by the following: 5:
s←rand(0,1)/rand(0,1)
 6:
$\alpha_{p}\leftarrow s\times max\left(position\right)/100$
 7:
$\alpha_{o}\leftarrow s\times max\left(orientation\right)/100$
 8:
$\alpha_{t}\leftarrow s\times max\left(torsion\right)/100$
 9:**for all** i ∈ position **do**10: **if** rand(0,1) $<P_{mutate}$ **then**11:  $x_{w}{\left[i\right]}^{\prime }\leftarrow x_{w}\left[i\right]+\alpha_{p}\times x_{d}\left[i\right]$12: **end if**13:
**end for**
14:**for all** j ∈ orientation **do**15: **if** rand(0,1) $<P_{mutate}$ **then**16:  $x_{w}{\left[j\right]}^{\prime }\leftarrow x_{w}\left[j\right]+\alpha_{o}\times x_{d}\left[j\right]$17: **end if**18:
**end for**
19:**for all** k ∈ torsion **do**20: **if** rand(0,1) $<P_{mutate}$ **then**21:  $x_{w}{\left[k\right]}^{\prime }\leftarrow x_{w}\left[k\right]+\alpha_{t}\times x_{d}\left[k\right]$22: **end if**23:
**end for**
24:Return $x_{w}^{\prime }$


### 3.4. Datasets

Two datasets of experimental protein–ligand complexes were used in this study. The PDBbind-CN database [22] is a manually curated database which provides a collection of known three-dimensional structures of protein–ligand complexes with experimentally measured binding affinity data. In each version of the database, three datasets are provided: the *general set* contains all valid complexes; the  *refined set* contains the good quality complexes (resolution ≤ 2.5 Å), which have only standard amino acid residues in the protein and common organic elements (i.e., *C*, *N*, *O*, *P*, *S*, *F*, Cl, Br, *I*, and *H*) in the ligand; and the *core set* contains three selected complexes with the highest, medium, and lowest binding affinities from each protein cluster generated from the refined set complexes. For example, the 2012 version of the PDBbind database (v2012) contains 2897 complexes in the refined set and 201 complexes in the core set. The core set is particularly suitable for validating protein–ligand docking methods owing to its use of a stringent selection procedure to ensure high quality and minimum redundancy in the final dataset. This dataset was used to train and test different variants of cuckoo search algorithms.

The second dataset used was the Astex Diverse set which is available from the Cambridge Crystallographic Data Center [23]. It contains 85 diverse protein–ligand complexes with a resolution better than 2.5 Å. The set was derived from different drug discovery studies, so all ligands are drug-like samples. This dataset has been widely used for protein–ligand docking method development, so it was used in this study to benchmark the performance of our method.

Both datasets were prepared by converting structure files into the PDBQT format using the python scripts prepare_receptor4.py and prepare_ligand4.py provided in the MGLTools package. Missing hydrogens were added, but nonpolar hydrogens were merged to the neighboring carbons based on the united-atom model scheme. Unrecognizable atoms were removed. For each protein receptor in the PDBbind dataset, the docking box was computed to include all atoms in the pocket residues file. For the Astex Diverse set, a standard cubic docking box of (22.5 × 22.5 × 22.5 Å) was used, following the setup in [11].

### 3.5. Search Space Coverage Metric

To investigate the behavior of a search process, we measured the search space coverage in an iteration as the sum of the distances between each pair of search agents. The *distance sum* (DS) was calculated as follows:

(1) $$DS=\sum_{i<j}\left|\right|X_{i}-X_{j}\left|\right|,\left(i,j\in \left[1,k\right]\right)$$

where *k* is the number of search agents. A large DS indicates that the search agents are more dispersed, implying that the search can cover the search space more thoroughly.

### 3.6. Statistical Tests

Given its capability to compare multiple methods, we used ANOVA with the post-hoc test of Tukey’s Honestly Significant Difference (HSD) to determine if Vina, PSOVina, and CuckooVina are different in terms of their docking performances. This post-hoc test is a comparison of means between pair of methods, taken into consideration the variances between all individuals within the methods. A significance level has been fixed at α = 0.05 in this study and the analyses were done using the R statistics package [24].

### 3.7. Implementation

CuckooVina was implemented based on the AutoDock Vina 1.1.2 by replacing the original Monte Carlo global search with CS and DE. The scoring function and the BFGS local search were not affected. All docking experiments were conducted on a desktop with Intel Core i7 2.93 GHz and 16 GB memory.

## 4. Conclusions

In this paper, we investigated the use of the cuckoo search (CS) algorithm for solving the protein–ligand docking problem. A hybrid method was proposed as a result, in which CS with a random walk for the mutation operation was used to perform local exploration of promising regions, while a modified DE process was used to perform global exploration throughout the entire search process. This new method, called CuckooVina, was shown to outperform AutoDock Vina in terms of binding affinity, RMSD, and success rate, as tested by two benchmark datasets. It predicts more accurate ligand binding poses with higher affinities than PSOVina with similar success rates. Although Vina and PSOVina are converged faster, their lower performances in binding affinity prediction suggest that both of them were trapped in local energy minima that led to premature convergences.

Our analysis of the distance sum of all search agents provided evidence that search agents in CS can maintain broader coverage of the search space, which is believed to be a crucial factor for its success. Unlike PSOVina where search agents can quickly collapse into limited search regions, in CuckooVina, by virtue of the modified DE process, diverse solutions are continuously generated. Once a new and better solution is identified (better than the better set), the region around this solution is immediately exploited. Therefore, a better balance between global search and local search is maintained in CS compared to PSO, contributing to its success in finding better docking solutions. An analysis of the effect of the number of search agents and the number of threads indicated that the search process in CuckooVina is robust, and reliable solutions can be obtained without the need of a large number of search agents and parallel threads. Our experiments suggest that the combination of eight search agents and eight to ten parallel threads yields optimal performance in CuckooVina. A future improvement to the CuckooVina algorithm would be the use of an adaptive approach for the switching parameter, $P_{worse}$, allowing the effort on global exploration to be adaptively adjusted based on the updating conditions.

## Figures and Tables

**Table 1:** Docking parameters used in experiments: For PSOVina, number of particles *N*, inertia weight ω, cognitive weight α, social weight β. For CuckooVina, number of host nests *k*, switching parameter $P_{worse}$, mutate probability $P_{mutate}$.

Method	Parameters
PSOVina	N=8; ω=0.36; $\alpha =\beta =0.99^{a}$
CuckooVina	k=8; $P_{worse}=0.25$; $P_{mutate}=0.25^{b}$

${}^{a}\;\omega ,\alpha$, and β were taken from [12]. ${}^{b}\;P_{worse}$ was taken from  [15] and $P_{mutate}$ was set equal to $P_{worse}$.

**Table 2:** Average performances of AutoDock Vina, PSOVina, and CuckooVina for docking the PDBbind 2012 dataset.

Method	RMSD (nm)	Success Rate (%)	Affinity (kcal/mol)	Run Time (s)	Convergence ${}^{a}$
AutoDock Vina	0.605	32.74	−7.645	13.73	0.48
PSOVina	0.409	49.12	−7.954	18.99	0.49
CuckooVina	0.364	51.08	−8.287	15.79	0.69

${}^{a}$ Fraction of energy evaluations reaching convergence.

**Table 3:** Average performances of AutoDock Vina, PSOVina, and CuckooVina for docking the Astex Diverse dataset.

Method	RMSD (nm)	Success Rate (%)	Affinity (kcal/mol)	Run Time (s)	Convergence ${}^{a}$
AutoDock Vina	0.272	59.84	−8.882	6.50	0.46
PSOVina	0.212	69.61	−8.952	8.99	0.48
CuckooVina	0.198	71.14	−9.153	7.06	0.67

${}^{a}$ Fraction of energy evaluations reaching convergence.

## Abbreviations

CS	cuckoo search
DE	differential evolution
PSO	particle swarm optimization
Vina	AutoDock Vina
